# Towards Quantum-Chemical Level Calculations of SARS-CoV-2 Spike Protein Variants of Concern by First Principles Density Functional Theory

**DOI:** 10.3390/biomedicines11020517

**Published:** 2023-02-10

**Authors:** Wai-Yim Ching, Puja Adhikari, Bahaa Jawad, Rudolf Podgornik

**Affiliations:** 1 Department of Physics and Astronomy, University of Missouri-Kansas City, Kansas City, MO 64110, USA; 2 Department of Applied Sciences, University of Technology, Baghdad 10066, Iraq; 3 School of Physical Sciences and Kavli Institute of Theoretical Science, University of Chinese Academy of Sciences, Beijing 100049, China; 4 CAS Key Laboratory of Soft Matter Physics, Institute of Physics, Chinese Academy of Sciences, Beijing 100090, China; 5 Wenzhou Institute, University of Chinese Academy of Sciences, Wenzhou 325000, China

**Keywords:** variants of concern, spike protein, RBD–ACE2 interface, mutational effect, atomic scale electronic structure, AABPU, partial charge, hydrogen bonding

## Abstract

The spike protein (S-protein) is a crucial part of the severe acute respiratory syndrome coronavirus 2 (SARS-CoV-2), with its many domains responsible for binding, fusion, and host cell entry. In this review we use the density functional theory (DFT) calculations to analyze the atomic-scale interactions and investigate the consequences of mutations in S-protein domains. We specifically describe the key amino acids and functions of each domain, which are essential for structural stability as well as recognition and fusion processes with the host cell; in addition, we speculate on how mutations affect these properties. Such unprecedented large-scale ab initio calculations, with up to 5000 atoms in the system, are based on the novel concept of *amino acid–amino acid-bond pair unit* (AABPU) that allows for an alternative description of proteins, providing valuable information on partial charge, interatomic bonding and hydrogen bond (HB) formation. In general, our results show that the S-protein mutations for different variants foster an increased positive partial charge, alter the interatomic interactions, and disrupt the HB networks. We conclude by outlining a roadmap for future computational research of biomolecular virus-related systems.

## 1. Introduction

The coronavirus severe acute respiratory syndrome coronavirus 2 (SARS-CoV-2) has taken millions of lives as of December 2022 [1]. This has galvanized different scientific communities to join forces in solving this pressing health problem. Several vaccines have been put forward in order to curb the proliferation of this virus, but its rapid mutation has generated new variants of concern (VOCs), such as Alpha, Beta, Gamma, Delta, and Omicron [2], that can enhance the SARS-CoV-2 virus transmissibility, infectivity, and antigenicity, thus restraining the effectiveness of these vaccines. Additional research is therefore urgently needed in order to maintain and extend the early success of the vaccines and keep them effective in controlling, and eventually stopping, the newly emerging VOCs of this pandemic.

**Figure 1:**
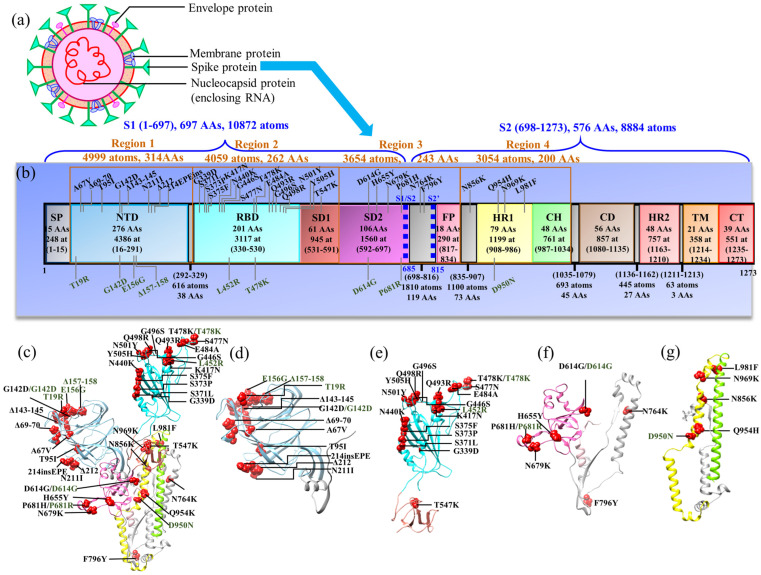
(**a**) Structure of SARS-CoV-2. (**b**) The schematic representation of the S-protein sequence in SARS-CoV-2 (ID: 6VSB) showing four regions of interest. The delta variant (DV) and Omicron variant (OV) are marked at the bottom and the top, respectively. The sequence numbers for the domains are—signal peptide (SP): 1–15; N-terminal domain (NTD): 16–291; receptor binding domain (RBD): 330–530; subdomain 1 (SD1): 531–591 and subdomain 2 (SD2): 592–697; fusion peptide (FP): 817–834; heptad repeat 1 (HR1): 908–986; central helix (CH): 987–1034; connector domain (CD):1080–1135; heptad repeat 2 (HR2): 1163–1210; transmembrane domain (TM):1214–1234; and cytoplasmic tail (CT):1235–1273. In addition, the number of amino acids and number of atoms (at) are marked for each domain. (**c**) Ribbon structure of the four regions in chain A of the S-protein. Ribbon structure of (**d**) region 1 including NTD, (**e**) region 2 (RBD–SD1), (**f**) region 3 including SD2–FP, and (**g**) region 4 including HR1–CH with mutations for Delta variant (DV) and Omicron variant (OV) marked by red spheres. The DV and OV are labeled by green and black, respectively.

SARS-CoV-2 belongs to the coronavirus family, which includes severe acute respiratory syndrome virus (SARS) and Middle East respiratory syndrome virus (MERS). Like other respiratory viruses, the coronavirus spreads through droplets discharged during breathing, coughing, sneezing, and speaking [3]. SARS-CoV-2 shares roughly 80% of its sequence with SARS-CoV-1 [4], and utilizes the same cellular entry receptor, angiotensin-converting enzyme 2 (ACE2) [5,6,7]. SARS-CoV-2 consists of four proteins: spike (S), envelope (E), membrane (M), and nucleocapsid (N) proteins, as shown in Figure 1a. Among these four proteins, the spike protein (S-protein) decorates the surface of the SARS-CoV-2 and initiates the human cell infection sequence by coming into contact with the ACE2 receptor.

The S-protein starts the viral entry and is therefore the main target for drugs, antibodies, and vaccine development [8,9,10,11,12,13]. It appears in a trimeric form and is composed of three chains either in up or down conformations. The chain with the up conformation is more important as it is receptor accessible [14]. Furthermore, each chain has two functional subunits—subunit S1 for receptor binding and subunit S2 for membrane fusion. The subunit S1 consists of N-terminal domain (NTD), receptor binding domain (RBD), subdomain 1 (SD1) and subdomain 2 (SD2). The subunit S2 consists of fusion peptide (FP), heptad repeat 1 (HR1), central helix (CH), connector domain (CD), heptad repeat 2 (HR2), transmembrane domain (TM), and cytoplasmic tail (CT). The schematic diagram of the S-protein showing its various domains is presented in Figure 1b, with all domains marked with the number of atoms and amino acids (AA) contained therein.

**Figure 2:**
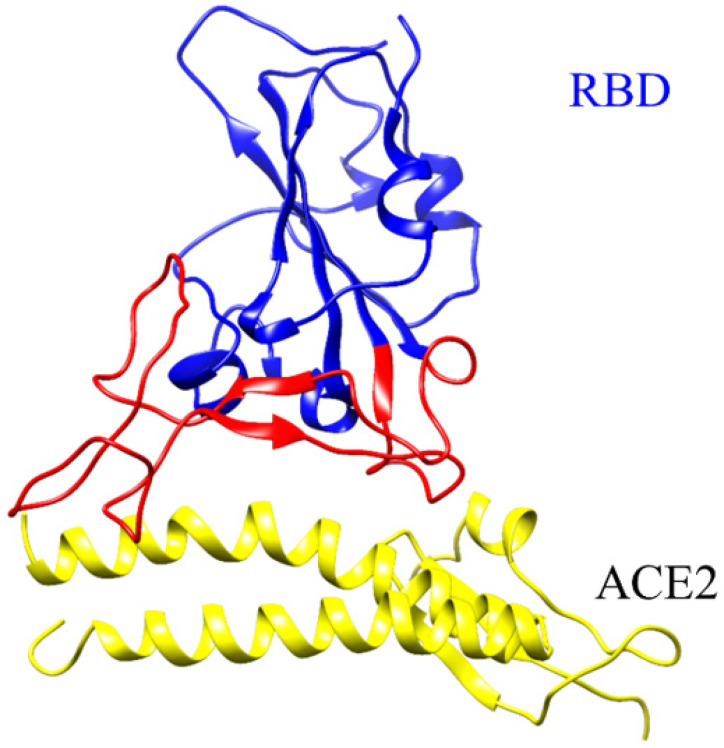
Interface of receptor binding domain (RBD) and angiotensin-converting enzyme 2 (ACE2). The red ribbon in the RBD is receptor binding motif (RBM).

The RBD from subunit S1 is receptor accessible and has initial interactions with the human ACE2 receptor. The interface between RBD and ACE2 is shown in Figure 2. In the RBD, the segment receptor binding motif (RBM) plays the key role in the interaction with ACE2. This interaction initiates a cascade of events that leads to the fusion of the viral membrane with the cell, enabling the virus entry.

Subunit S2 is responsible for the fusion process itself, which involves the protein cleavage at S1/S2 and S2′ sites [15]. These sites are marked in Figure 1b with blue dashed lines. The S1/S2 cleavage site is located at the boundary between the S1 and S2 subunits and displays a unique polybasic insertion of furin recognition site _681_PRRAR|S_686_| ( denotes the proteolytic cleavage site) [16]. This unique polybasic insertion S1/S2 site is considered as the reason behind its high infectivity and transmissibility [16,17]. The S2′ cleavage site, also known as the transmembrane serine protease 2 (TMPRSS2) cleavage site, lies immediately upstream of the S2 subunit fusion peptide (FP) domain. The S-protein must be sequentially cleaved at these S1/S2 and S2′ sites to activate the cleavage process and mediate cell–cell fusion, which is a complicated mechanism [18]. In brief, after the RBD of the S1 subunit recognizes and attaches to the ACE2 receptor, the S-protein is initially cleaved by the protease furin at the S1/S2 junction, with both S1 and S2 remaining non-covalently associated in the prefusion conformation [15,19,20]. Then, a second cleavage by TMPRSS2 at the S2′ site is induced, in which the S-protein undergoes significant conformational changes, resulting in the dissociation of S1 and the irreversible refolding of S2 into a postfusion structure [18]. This causes the virus and host cell membranes to fuse, allowing infection to begin.

The details of the structure of the S-protein are inferred from the Cryo-electron microscopy (cryo-EM) as protein data bank (PDB) IDs 6VSB [14], 6VYB [18], and 6VXX [18]. Based on these structural details, many computational studies were performed on the S-protein by using different flavors of the molecular dynamics (MD) simulation. For example, a full-length model of the glycosylated SARS-CoV-2 S-protein has been built and simulated using this MD approach to explore its dynamic and structural insights [21], the role of glycans in its functions [22] or in facilitating the transitions from “closed” to “open” RBD conformation [23], as well as determining its intermediate state structures in the opening pathway [24]. Besides that, countless MD studies have been conducted using these cryo-EM structures as well as the additional hundreds of structures that have been deposited in the PDB using either cryo-EM or X-ray techniques. In contrast, and surprisingly, very few computational studies have been performed based on the ab initio methodologies. In order to upend this dissonance, we have conducted different ab initio calculations, mostly on wild-type, Delta and Omicron variants, focused on several domains of the S-protein [25,26,27,28,29,30,31], RBM–ACE2 and RBD–ACE2 interface [32,33,34] as well as miniproteins [35]. Moreover, we have combined the ab initio calculations with molecular dynamics for the S-protein–ACE2 interface in order to get a clearer and more accurate picture of their recognition process when forming a complex [32].

This paper covers ab initio studies of domains from NTD to CH in the S-protein and RBD–ACE2 interface as well as some drug designs. It should be indicated that it is currently impossible to perform extremely large-scale ab initio all-atom computations of the whole S-protein of around 45,000 atoms. We have used a *divide* and *conquer* strategy by focusing on individual structural domains of only the up-conformation chain of the S-protein [27]. With this strategy, it is possible to treat each domain as an independent model and connect the results in an insightful way for the entire S-protein (see next section). The ribbon structure of the S-protein, including the domains from NTD to CH, are divided into four regions, are shown in Figure 1c–g. The Delta variant (DV) and Omicron variants (OV) BA.1 are marked in the ribbon structure of the four regions of interest. Our overall focus will be the bonding and partial charge. In what follows, WT stands for wild type, DV for Delta variant, BV for Beta variant, and OV for Omicron variant respectively.

## 2. Computational Models and Methods

### 2.1. Modelling Structures from Various PDBs

Ab initio quantum chemical calculations are not feasible for exceptionally large systems with hundreds or thousands of atoms, including the whole S-protein. We have therefore used the divide-and-conquer strategy to design our computable initial model. In the divide-and-conquer strategy, we partitioned the S-protein domains from NTD to CH into four regions: region 1, region 2, region 3, and region 4, including domains NTD, RBD–SD1, SD2–FP, and HR1–CH, respectively. 

In our initial calculations [25,26,27], the models of S-protein domains were prepared using Cryo-EM 3D-structure of SARS-CoV-2 with a 3.5 Å resolution, as deposited with PDB ID 6VSB by Wrapp et al. [14]. The chain A with the up-conformation, corresponding to a receptor-accessible state, was selected for the modelling. The S-protein from the 6VSB structure misses some flexible segments of amino acids (AAs) due to technical difficulties, but Woo et al. [36] later provided a more complete structure of 6VSB. In our recent publications on the study of domains/region of interests [29,30,31], we thus used 6VSB 1_2_1 from Woo et al. In our initial studies [25,26,27], we added hydrogen atoms using the UCSF Chimera [37], but later [29,30,31,32,33] switched to the LEaP module from the AMBER (Assisted Model Building with Energy Refinement) package [38,39]. In fact, our experience indicates that AMBER is more accurate in adding the H atoms, especially for charged residues, by using template-specific force fields and depending on the protonated state of these residues. In all regions except region 1, the WT model was used as a template to generate the corresponding variant model. Specifically, the DV and OV BA.1 with all mutations, marked in Figure 1b, were prepared based on their initial WT model by substituting the certain WT residue(s) to mutated residue(s) using the Dunbrack backbone-dependent rotamer library [40] from USCF Chimera [37]. For example, aspartic acid at position 614 was substituted by the glycine to generate the D614G mutation in region 3 for both DV and OV.

For region 1, PDB IDs 6VSB_1_2_1, 7SBL [41] and 7TGW [42] were used for WT, DV, and OV, respectively. Both PDB IDs 7SBL and 7TGW were obtained using electron microscopy with a resolution of 3.40 Å and 3.00 Å, respectively. Region 1, which includes the NTD domain, contains AAs from the sequence ranging from V16 to F329. This domain has T19R, G142D, E156G, and Δ157–158 mutations for DV and A67V, Δ69–70, T95I, G142D, Δ143–145, N211I, Δ212, and 214EPEins mutations for OV BA.1. Both variants have G142D in common. NTD has a few deletions and a few insertions of AAs in its variants, implying differences in the total number of AAs, with 314, 312, and 311 AAs for WT, DV, and OV, respectively. The total number of atoms are 4999, 4962, 4954, for WT, DV, and OV, respectively. These are the largest regions that allowed us to perform detailed ab initio computations for the S-protein.

For region 2, which includes domains RBD and SD1, the PDB ID 6VSB_1_2_1 was used to prepare WT, DV, and OV models. The AA sequence selected for region 2 ranges from P330 to S591 with 262 AAs. The RBD domain of DV has two mutations—L452R and T478K. Similarly, OV BA.1 has 15 mutations—G339D, S371L, S373P, S375F, K417N, N440K, G446S, S477N, T478K, E484A, Q493R, G496S, Q498R, N501Y, and Y505H. Both DV and OV have the T478K mutation in common. Domain SD1 has no mutation in DV and one mutation (T547K) in OV. Region 2 consists of 4059, 4072, and 4123 atoms for WT, DV, and OV, respectively.

Region 3 contains SD2 and FP domains, including both cleavage sites S1/S2 and S2′, crucial for the fusion process. PDB ID 6VSB_1_2_1 was used to prepare WT, DV, and OV models. Region 3 consists of 243 AAs, i.e., from F592 to I834. DV consists of two mutations (D614G and P681R) and OV BA.1 consists of six mutations—D614G, H655Y, N679K, P681H, N764K, and F796Y. The WT, DV, and OV models have 3654, 3659, and 3681 atoms, respectively.

Region 4 includes domains HR1 and CH. This region was prepared using PDB ID 6VSB_1_2_1. It consists of 200 AAs. The DV has one mutation (D950N) and OV BA.1 has four mutations—N856K, Q954H, N969K, and L981F. The WT, DV, and OV models have 3054, 3056, and 3071 atoms, respectively.

To model the interface between the S-protein and ACE2, we designed an entire RBD with a portion of ACE2 or only its RBM with the same portion of ACE2. The interface model was prepared using PDB ID 6M0J [7], which was obtained by using x-ray diffraction with resolution of 2.45 Å. In the RBM–ACE2 interface model, RBM contains 71 AAs from S438 to Y508 and ACE2 contains 117 AAs from S19 to I88 (70 AAs of α1 and α2 motifs), in addition to G319 to T365 (47 AAs of β3 and β4 motifs). The RBM is the main functional motif of RBD that interacts with ACE2. The segment of ACE2 we selected includes all interacting AAs according to the high-resolution crystal structure information [7,43]. We added Na^+^ ions to further neutralize the system via a Coulomb potential on a grid, using the LEaP program in the AMBER package [39]. In addition, hydrogen atoms were added using the LEaP module [38,39]. We performed a comparative study of the RBM–ACE2 interface models for SARS1 [32], SARS2 WT [32,33], BV [32], and OV [33] models with 2942, 2930, 2942, and 2964 atoms, respectively.

The Beta variant (BV) has three mutations in the RBM (K417N, E484K, and N501Y), which were prepared using the Dunbrack backbone-dependent rotamer library [40] from USCF Chimera [37]. OV BA.1 has 10 mutations in the RBM (N440K, G446S, S477N, T478K, E484A, Q493R, G496S, Q498R, N501Y, and Y505H), which were also prepared using the same approach [33]. The SARS1 model was prepared using PDB ID 2AJF [44], which was obtained from x-ray diffraction with a resolution of 2.90Å. The RBM–ACE2 interface model consists of the same number of AAs for ACE2, i.e., S19 to I88 and G319 to T365, respectively, whereas the 70 AAs for RBM have sequence numbers from T425 to Y494.

In the RBD–ACE2 interface model, PDB ID 6M0J [7] was used for WT and PDB ID 7WBP [45] for OV. PDB ID 7WBP was obtained using x-ray diffraction with a resolution of 3 Å. The OV BA.1 RBD contains fifteen mutations (G339D, S371L, S373P, S375F, K417N, N440K, G446S, S477N, T478K, E484A, Q493R, G496S, Q498R, N501Y, and Y505H). The AA sequence for ACE2 is the same as in the RBM–ACE2 interface model with 117 AAs, whereas the RBD consists of a larger number of AAs in the sequence T333-G526 (194 AAs). This RBD–ACE2 interface model has 311 AAs. The WT and OV RBD–ACE2 interface models have 4817 and 4873 atoms, respectively.

### 2.2. Ab Initio Computational Packages

*Vienna ab initio simulation package* (VASP) [46], a density functional theory (DFT)-based package, is well known for its accurate optimization of complex materials. VASP uses the concept of pseudopotential approximation instead of exact potential. This feature ignores the core level nodal features but emphasizes the most important region that forms bonds between two atoms. We used VASP for geometric optimization, which is the first and most important step. This step provides the accurate structure that will be used for further calculations. In VASP, we used the projector augmented-wave (PAW) [47,48] method with Perdew–Burke–Ernzerhof (PBE) [49] exchange correlation functional within the generalized gradient approximation (GGA). PBE is one of the best GGAs available in VASP.

Our complex models are large and expensive for ab initio simulations. Based on our experience, we used an energy cut-off of 500 eV with electronic convergence of 10^−4^ eV, force convergence for ionic relaxation to −10^−2^ eV, and a single k-point sampling.

The optimized structure from VASP is used as the input for *orthogonalized linear combinations of atomic orbitals* (OLCAO), an in-house-developed package. OLCAO [50] is also based on DFT, and its combination with VASP works very well for many complex materials [51,52,53,54,55,56,57,58,59], as well as for biomolecules such as the S-protein [25,26,27,29,30,31,32,33,35]. OLCAO uses atomic orbitals for basis function expansion. It is used to calculate electronic properties such as total and partial density of states (TDOS/PDOS), optical properties, effective charge ($Q^{*}$), and bond order (BO).

OLCAO uses Mulliken’s population analysis to calculate effective charge ($Q^{*}$) and bond order (BO). $Q^{*}$ is the number electronic charges associated with the atom, defined as

(1) $$Q_{\alpha }^{*}=\underset{i}{\sum }\underset{n.occ}{\sum }\underset{j,\beta }{\sum }C_{i\alpha }^{*n}C_{j\beta }^{n}S_{i\alpha ,j\beta }$$

Here $Q_{\alpha }^{*}$ is the effective charge on atom α. The deviation of effective charge from the neutral charge is the partial charge $\Delta Q_{\alpha }$, $\Delta Q_{\alpha }=Q_{\alpha }^{0}-Q_{\alpha }^{*}$. Here, $Q_{\alpha }^{0}$ is the charge on the neutral atom α.

BO is the overlap population $\rho_{\alpha \beta }$ between pair of atoms (α,β), defined as

(2) $$\rho_{\alpha \beta }=\underset{n,occ}{\sum }\underset{i,j}{\sum }C_{i\alpha }^{*n}C_{i\beta }^{n}S_{i\alpha ,j\beta }$$

where $S_{i\alpha ,j\beta }$ are the overlap integrals between the $i^{th}$ orbital in $\alpha^{th}$ atom and the $j^{th}$ orbital in $\beta^{th}$ atom, and $C_{j\beta }^{n}$ are the eigenvector coefficients of the $n^{th}$ band, $j^{th}$ orbital in the $\beta^{th}$ atom. The BO determines the strength of the bonds, and summing the BO of all bonds formed inter-amino acids gives the *amino acid*–*amino acid bond pair* (AABP) discussed next.

### 2.3. Key Concepts AABP and AABPU

As mentioned above, the bond order values $\rho_{\alpha \beta }$ obtained for each pair of atoms from the OLCAO package can be further generalized to obtain the bond order between groups of amino acids (AAs). The bond order for inter-amino acids has been named as *amino acid*–*amino acid bond pair* (AABP). AABP sums all bond orders formed between two amino acids u and v:

(3) $$AABP\left(u,v\right)=\underset{\alpha \epsilon u}{\sum }\underset{\beta \epsilon v}{\sum }\rho_{\alpha i,\beta j\;}$$

Here the summations are over atoms α in AA u and atoms β in AA vs. AABP as introduced above and, based on the quantum mechanical analysis of OLCAO, is a novel and rigorous tool. AABP accounts for all possible bonding between AAs, including the hydrogen bonding, and determines the strength of the bonds formed between whole amino acids. AABP for the selected site or selected AAs includes all inter-AAs bonding formed with the selected AA. AABP can be further resolved into nearest neighbor (NN) AAs and non-local (NL) AAs. NN AAs in the protein sequence yield a higher contribution to the bonding. However, it is the NL AAs that dictate the 3D protein structure, with all the twists and turns, that can further help identify the shapes and possible functionality of proteins. In addition, we can also identify the contribution of *hydrogen bonds* (HB) to the overall AABP. Even though the strength of HBs is relatively weaker compared to the covalent bonds, their number is large enough to have an impact on the bonding between AAs.

From AABP we have further designed *AABP units* (AABPU), composed of several interacting amino acids as a unit. In general, all amino acids are linked with others in some way, but we specifically focus on selected mutated amino acids in the sequence and identify its NN and NL AAs, grouping them together as an AABPU. AABPU thus represents a type of collective order parameter and a fundamental new structural unit specifically in proteins, but also in general biological materials. The AABPU is particularly helpful in studying mutations and the ongoing new variants of SARS-CoV-2, characterized by either replacement of certain AAs or their outright deletion. In this context, AABPU can detect and quantify significant changes in the overall structure and the nature of bonding between different AAs.

## 3. Results

### 3.1. Structure Domains in Four Regions of S-protein (NTD, RBD–SD1, SD2–FP, and HR1–CH)

The subunit S1and S2 domains are divided into four regions as discussed below:

**Region 1 (NTD):** Structurally, the NTD is composed primarily of four stacked β-sheets and a number of connecting flexible loops (Figure 1d) containing several N-linked glycans [60]. The exact role of NTD as a functional unit is unknown [60,61]. However, there is evidence that NTD might play an important role in facilitating S-protein’s prefusion-to-postfusion transition [62,63], serve as a critical epitope for neutralizing antibodies [64,65,66], and contribute to infection and cell–cell fusion [61,67]. Intriguingly, it has been demonstrated that the NTD allosterically regulates the S1/S2 cleavage and spike-mediated functions [61,63].

NTD has mutations in both DV (T19R, G142D, E156G, and Δ157–158) and OV (A67V, Δ69–70, T95I, G142D, Δ143–145, N211I, Δ212, and 214EPEins). Besides substitution, these mutations have several deletions as well as insertions of AAs. Both DV and OV have G142D in common. In Table 1, we show the AABP values for the substituted AAs of both DV and OV. The total AABP values include all inter-AA bondings of the selected AA or mutated site. This value can be further divided into the contributions from NN and NL AAs, and the contribution from the HBs can be identified directly. There is a complex reciprocity in every mutation, and combination of several mutations can impact the dynamics of mutation. For example, G142D in DV has only four NL AAs, whereas in OV, it has nine NNs. In OV, V143 to Y145 are deleted and H146 becomes the new NN of D142, changing the dynamics completely. D142 of DV has a lower total AABP value, whereas the total AABP value of D142 in increases in comparison to WT. This implies that the AABPU of DV D142 is weaker and that of OV D142 is stronger than WT G142. The three DV substitutions listed in Table 1 have a decrease in total AABP value in comparison with WT. In the case of OV mutations, it has both an increase and decrease of AABP value in comparison with WT, which denotes the gain in strength and loss in strength of the unit, respectively.

Previous evidence has observed that NTD mutations in the N3 loop (residues 140–156) and the N5 loop (residues 246–260) induce immune evasion [68,69]. On the other hand, NTD deletions have been characterized as manipulating NTD antigenicity, especially Δ143–145 [70]. Additionally, Δ69–70 is predicted to alter the conformation of an exposed NTD loop and has been reported to be associated with increased infectivity [71]. The detailed results comparing WT, DV, and OV for NTD and the effect of deletion will be published in the future.

As we will see later, one major limitation of using ab initio calculations is that they are restricted to small models of thousands of atoms, making it currently impossible to reveal the consequences of mutations on other S-protein chains or antibodies. So here, we only conjecture that these NTD mutations and deletions could alter the interchain and/or interdomain interactions of the S-protein as well as conferring the antibody resistance, affecting its structure, flexibility, dynamics, and antigenicity.

**Region 2 (RBD**–**SD1):** Region 2 consists of domains RBD and SD1. RBD is considered the most important domain as it is the first to be in contact with the human cell ACE2. Structurally, the RBD is divided into a core RBD and a receptor-binding motif (RBM). The core RBD structure is composed of a five-stranded antiparallel β- sheet that is covered on both sides with short connecting α- helices, while the RBM is formed by an extended loop that wraps around one edge of the core structure and interacts directly with the receptor ACE2, forming the interface [7,60]. Hence, the RBD is used as the principal target for drugs and vaccine development [6,34,35,72,73]. Specifically, the RBD is immunodominant and contains epitopes for 90% of the antibodies elicited by natural infection or vaccination [74].

The enrichment of mutations in key RBD or RBM residues across VOCs have a significant impact on the interaction with ACE2 and antibodies, either positively or negatively. Since SARS-CoV-2 is continuously evolving, many VOCs are still emerging with a significant number of mutations in the RBD. Specifically, there are only 2 mutations in DV, but there are 15 mutations in OV BA.1 (G339D, S371L, S373P, S375F, K417N, N440K, G446S, S477N, T478K, E484A, Q493R, G496S, Q498R, N501Y, and Y505H), of which 12 (G339D, S373P, S375F, K417N, N440K, S477N, T478K, E484A, Q493R, Q498R, N501Y, and Y505H) are also seen in another OV (BA.2) and 11 (G339D, S373P, S375F, K417N, N440K, S477N, T478K, E484A, Q498R, N501Y, and Y505H) in OV BA.5. In OV BA.5, one mutation (L452R) is common with DV. Hence, a detailed study of DV and OV BA.1 could provide information on the biological function of mutations as well as pave the route for the prediction of new variants. These rapidly occurring mutations change the binding to ACE2 and will be further discussed in Section 3.2. So far, we have modeled the RBD with SD1 as region 2. SD1 acts as a hinge point for the RBD up/down conformation transitions, with support from the NTD and SD2 [75,76]. SD1 is located below the RBD and rotates with the RBD in the transition to a receptor-accessible state [60]. Structurally, it is formed by β- structures. SD1 does not have any mutations in DV but has one mutation, T547K, in OV BA.1.

We provide AABPU analysis (Table 2) for 18 mutations sites, among which two are from DV and 16 are from OV BA.1 [31]. From the AABPU analysis, we can identify stronger units and see the changes introduced by mutations such as the change in volume and area, partial charge, and overall bonding. The overall bonding includes contributions from NN or NL AAs as well as contributions from the HBs.

**Figure 3:**
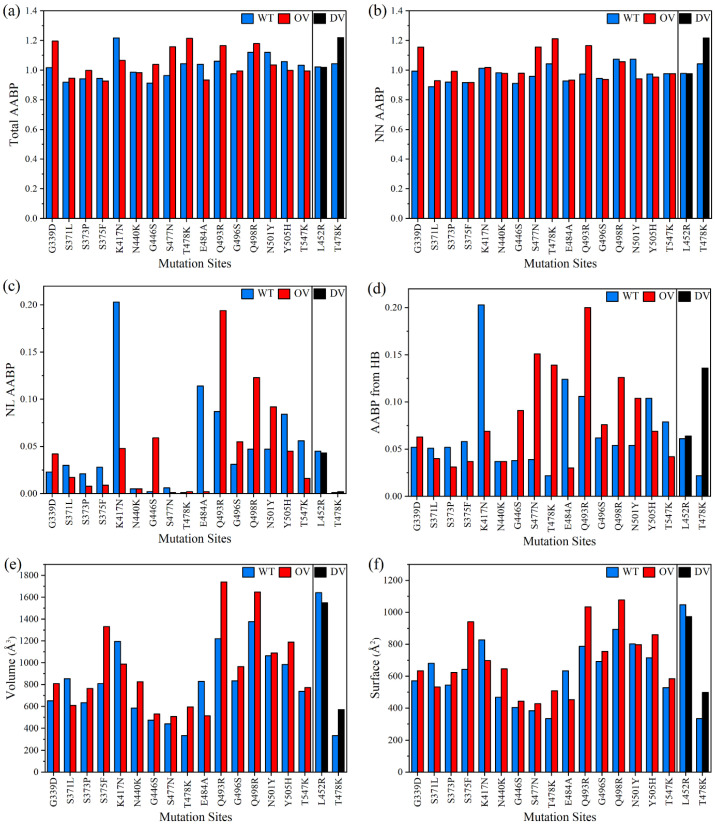
Comparison of 2 DV and 16 OV mutations with their corresponding WT sites in the RBD–SD1 model (from reference [31]) in terms of: (**a**) Total AABP; (**b**) NN AABP; (**c**) NL AABP; (**d**) AABP from HB; (**e**) Volume; and (**f**) Surface area.

From Table 2, it can be identified that NN AAs have the highest influence, with NL AAs and HBs also playing a significant role. Besides the contribution of bond strength, the number of bonds is also important. Mutations can affect the AABP by influencing bond strength as well as the number of bonds, mostly due to the change in the number of NL AAs. For example, Q493R exhibits an increase in total AABP (TAABP) after mutation, with a significant contribution from NL AAs as well as from HBs. OV R493 has the highest difference in the number of HBs involved, displaying a substantial change in the intramolecular HB distribution. The TAABP increases as well as decreases after the mutation, showing dynamic changes for each mutation site in DV and OV BA.1. Figure 3 taken from our publication [31] shows the overall change in the total AABP values with the contribution from NL and NN AAs, in addition to the overall contribution from HBs. The range of AABP values in NL and HB are similar, implying that most of the NL bonding is from HBs which, nevertheless, also contributes to the NN bonding.

Other parameters such as the volume and area of the unit also change due to the changes in the number of NL AAs. These changes in the volume and surface of the AABPU are also shown in Figure 3e,f. In general, mutations also increase the volume, except for DV L452R, and OV S371L, K417N, and E484A.

**Figure 4:**
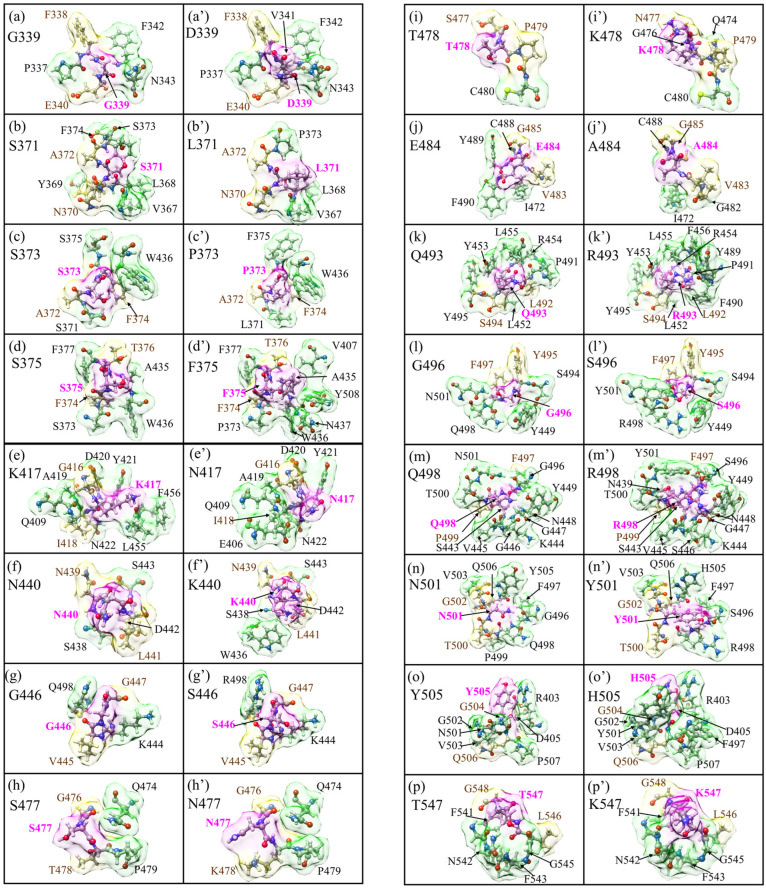
Details of the shape change of AABPU of the sixteen mutation sites in RBD–SD1( from reference [31]): (**a**) G339; (**b**) S371; (**c**) S373; (**d**) S375; (**e**) K417; (**f**) N440; (**g**) G446; (**h**) S477; (**i**) T478; (**j**) E484; (**k**) Q493; (**l**) G496; (**m**) Q498; (**n**) N501; (**o**) Y505; and (**p**) T547 for the WT. (**a’**) D339; (**b’**) L371; (**c’**) P373; (**d’**) F375; (**e’**) N417; (**f’**) K440; (**g’**) S446; (**h’**) N477; (**i’**) K478; (**j’**) A484; (**k’**) R493; (**l’**) S496; (**m’**) R498; (**n’**) Y501; (**o’**) H505; and (**p’**) K547 for the OV. The surface of mutated sites is shown in magenta, and the surface of NN and NL are shown in yellow and green, respectively. All NN and NL AAs are marked near to their surface in brown and black, respectively.

All AAs interacting with the central AAs, or the selected site, are considered as an AABPU of the selected site. The visual representation of these units can be seen in Figure 4, taken from our publication [31], and shows the changes in the AABPU for 16 mutations of OV BA.1 in comparison with WT. These figures show the changes in the positioning as well as number of NL AAs. In addition, this figure provides an idea of the shape and volume of the unit as well as the changes due to the mutation. 

The largest total AABP value comparing WT and OV BA.1 is in WT K417 and exhibits a significant contribution from the NL AAs. The overall higher TAABP denotes stronger bonding within the defined unit and vice versa. Each mutational site is associated with respective changes in the TAABP, and our results can further identify the source for stronger as well as weaker TAABP. It must be pointed out that this analysis is for the prefusion structure of RBD–SD1 and the dynamics changes in the interface region discussed in Section 3.2.

Partial charge (PC) determines the electrostatic potential of a biomolecule, which is important for predicting the overall long-range intermolecular interactions [77]. Using the OLCAO package, the PC for every atom in the system can be calculated by using ab initio methods, and can be useful for computing electrostatic interactions using, e.g., the Delphi software [32,78]. It could just as well be used to improve the accuracy of the PCs used in most of the MD simulations. The partial charge for the AABPU is denoted by PC* and is calculated by summing the partial charges of all AAs or summing the partial charges of all the atoms involved in the AABPU. Out of the 16 OV BA.1 mutations, 10 have positive PC*, and both DV mutations have an increase in positive PC*. Only the three OV BA.1 mutations (G339D, K417N, and Q493R) become more negativly charged. The more substantial increase in positive PC* of OV BA.1 mutations are observed in G446S, S477N, T478K, Q498R, N501Y, and T547K. The overall increase of PC* in the positive direction after mutation could indicate a change in surface charge distribution affecting the unspecific electrostatic interaction with predominantly negatively charged host cell membrane as well as the specific ACE2 or antibody binding [34,79].

**Region 3 (SD2**–**FP):** Region 3 includes domains from SD2 to FP. Structurally, SD2 is formed by two stacked β-sheets, each containing four strands [60]. FP is a short segment of 15–20 conserved amino acids from the viral family, primarily composed of hydrophobic residues such as glycine (G) or alanine (A) that anchor to the target membrane when the S-protein adopts the pre-hairpin conformation [80]. It plays a central function in mediating cell–cell membrane fusion [81]. Besides these two domains, this region consists of cleavage sites S1/S2 and S2′. The SD2 connects subunits S1 and S2, so the first cleavage site S1/S2 falls within it and the second cleavage site S2′ is located just before the start of FP. The furin cleavage site S1/S2 is not observed in SARS but is observed in all SARS-CoV-2 genomes and is considered to be very important. This cleavage helps the viral entry into human cell and needs to occur before viral fusion [82].

There are two mutations in DV (D614G and P681R) and six mutations in BA. 1 OV (D614G, H655Y, N679K, P681H, N764K, and F796Y). The six mutations in BA.1 OV are common to BA.2, BA.2.75, BA.3, BA.4, and BA.5. Their study can help understand upcoming newer variants. The mutation D614G is common to both DV and OV variants. D614G is considered to promote the open-up conformation of RBD, making it receptor accessible [83]. The DV mutation P681R and OV mutation P681H are right at the polybasic insertion–furin recognition site _681_PRRAR|S_686_. P681R is known to enhance cleavage [84,85,86,87], and both P681R and P681H are considered as important factors for its infectivity [86,87].

The AABP results for the SD2–FP region comparing WT, DV, and OV are shown in our previous works [29,30]. There is a decrease in total AABP values of the D614G unit when comparing WT (0.917 e^−^) with both DV (0.901 e^−^) and OV BA.1 (0.908 e^−^). This decrease in total AABP value is due to several reasons, such as a decrease in the number of NL AAs, which results in a decrease in the overall bond strength in the AABPU. This decrease of AABP value can predict the enhancement of the flexibility of the AAs for easy twisting and turning. When D614 is substituted by G, it loses the sidechain, resulting in a reduction of intramolecular interactions in the same protomer. Our result identifies the disruption caused in the NL network that could lead to a promotion of the up-conformation of the S-protein, resulting in a cleavage enhancement as disclosed by Zhang et al. [83] and Gobeil et al. [19]. In addition, there is a slight increase in the contribution of HB AABP of the D614G unit when comparing WT (0.040 e^−^) [29,30] with both DV (0.041 e^−^) [29] and OV BA.1 (0.042 e^−^) [30]. The increase in HB is consistent with Raghav et al. [88], who report enhanced binding of the S-protein with TMPRSS2 in the D614G mutation.

In the case of P681R of DV, there is an increase in the number of NL AAs, but the total AABP value still decreases due to a decrease in bond strength with both NN AAs and NL AAs. P681H of OV BA.1 has the same number of NL AAs but also has a lower total AABP value in OV BA.1 due to a decrease in bond strength of NN AAs and NL AAs. Both mutations (P681R and P681H) result in a decrease in the total AABP value, which could be a reason behind its high infectivity. Mutation N679K, located close to the furin cleavage region, also exhibits a decrease in the total AABP value and is known to display an increase of the furin cleavage of OV [89]. Some studies have observed that N679K and P681H do not necessarily enhance the cleavage [79,87,90,91,92], but they may still help OV for higher infectivity.

PC* for P681R in DV [29] and six mutations in OV BA.1 [30] increase in a positive direction in comparison with WT. The 681 site is located adjacent to the furin cleavage site. Mutations at this site have been reported to play a significant role in the cleavage process [29,84,87,93]. Mutations R681 of DV and H681 of OV are known to enhance the infectivity, and both exhibit an increase in the positive PC*. In fact, positive charge is required for the host furin-like proteases to cleave the S-protein [87]. However, some studies suggest no protein cleavage enhancement by P681H and N679K [79,90,91,92]. This suggests that additional mutations near the furin cleavage site may interfere with its cleavage.

We believe that the decrease in total AABP value of the D614G, P681R, and P681H units could promote the turning and twisting of AAs that could facilitate the RBD to move into the open-up conformation and be receptor accessible. In addition, the 681 site has a proline in WT, known for its rigidity, and its mutation leads to a decrease in its rigidity. Additionally, our result on PC yields support for R681 to take part in S1/S2 cleavage enhancement as shown in human airway epithelial cells [86].

**Region 4 (HR1**–**CH):** Region 4 includes the domains HR1 and CH. In the prefusion conformation, subunit S1 wraps around subunit S2, forming a central helical bundle with HR1 bending towards the viral membrane [60]. HR1 goes under drastic conformation transition leading to insertion of FP in the target membrane [60]. HR1 with other residues from 758 to 784 coils to contribute to the stability of the S-protein [60]. HR1 interacts with HR2 in forming six helical bundles, bringing the viral and host membrane closer for fusion [94]. HR1 is highly conserved among SARS [95], and, consequently, this region has been a target [96,97,98] for antibodies. A pan-coronavirus fusion inhibitor EK1, aimed at HR1, was found to inhibit membrane fusion by SARS-CoV-2 and MERS-CoV [99]. Between HR1 and CH there is a transitional bend that, when fixed with two consecutive proline residues, blocks S-protein conformation from the prefusion to the postfusion state. This leads to stabilization of the S-protein and is important for vaccine development [100,101,102].

In Region 4, there is one DV mutation (D950N) and four OV BA.1 mutations (N856K, Q954H, N969K, and L981F). Mutations Q954H and N969K are present in the newer OV BA.2 and BA.4. The AABP values are shown in our past publications [29,30]. Even with the same number of NL AAs, the total AABP value for D950N decreases due to lower contribution from NN AAs, NL AAs, and HB [29]. According to mutation mapping, it is suggested that the D950N mutation may contribute to a modulation of the S-protein dynamics, similar to D614G [103]. The 981 site is far from the cleavage site S2′ but is near the prefusion-stabilizing two-proline mutations (K986P and V987P) utilized by *Pfizer-BioNTech* and *Moderna* for vaccine development [104,105]. There is an increase in total AABP value after mutation in L981F, even with a decrease in the number of NL AAs. Even though the number of interactions is fewer, the interactions after mutation are stronger and thus lead to higher contributions from the NL AAs and HBs. In Q954H, the total AABP value slightly decreases after mutation. However, this site has an overall higher contribution from NL AAs in comparison to other mutations nearby. N969 and L981F have an increase in total AABP value after mutation. As these mutations are in HR1, we hypothesize that they may play a role in interaction between HR1 and HR2, possibly enhancing the six-helical bundle, bringing the viral and the host membrane closer for better fusion and higher infectivity [80].

PC* for D950N of DV and three OV mutations (N856K, H969K, and L981F) increases positively. The 856 site is closer to S2′ and it may play a role for the cleavage process in the S-protein. In addition, mutations N764K (region3), N856K, and N969K from region 4 form interprotomer electrostatic contacts with the neighboring protomers enhancing the stability of the S-protein [106].

### 3.2. RBD–ACE2 Interface Complex

The recognition process between the S-protein RBD and the ACE2 receptor is the initial stage of viral infection, which is critical in determining host cell and tissue tropism [18]. Further, the virus-cell membrane fusion process is induced when this association event occurs [14]. Indeed, the binding affinity between the RBD and ACE2 as well as the cleavage process both contribute significantly to SARS-CoV-2 infectivity and transmissibility [18,107]. These critical functions of the RBD–ACE2 complex indicate that it is a primary target for developing many therapies such as vaccines, antibodies, and small inhibitor drugs. Consequently, extensive efforts towards targeting this interface complex culminated in the development of many vaccines and antibodies in a short period of time, as mentioned earlier.

However, the emergence of many VOCs with dozens of RBD mutations, such as the OV, represents one of the major current challenges of these therapeutic strategies related to the COVID-19 pandemic. In an attempt to understanding the consequence of RBD mutations on the ACE2 binding, we investigated the RBM–ACE2 complex in SARS-CoV-1 (SARS1), SARS-CoV-2 (SARS2 WT), Beta (BV), and Omicron (OV), as well as the RBD–ACE2 complex in WT and OV [31,32,33].

**Figure 5:**
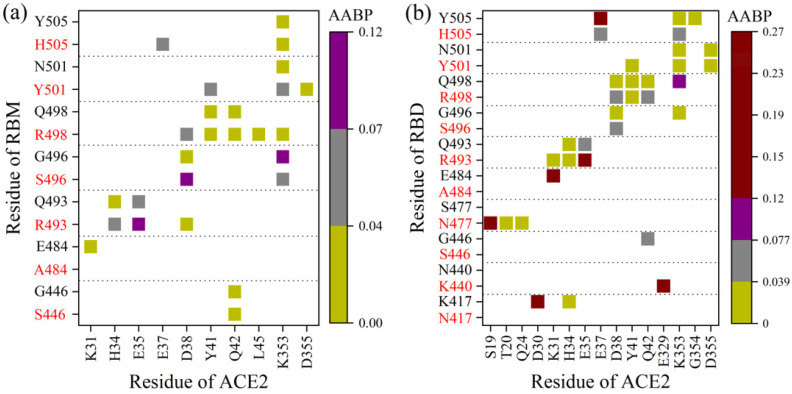
AABP pair map for (**a**) RBM–ACE2 (from reference [33]) and (**b**) RBD–ACE2 (from reference [31]) for only the mutated OV residues (red characters on y-axis) that form pairs with ACE2, compared with their WT counterparts (black). Each square cell represents one pair for the intersection AA from RBM/RBD on the vertical axis and AA from ACE2 on the horizontal axis. These pairs have different strengths based on the AABP value.

Figure 5a compares the AABP interacting pairs between the RBM and ACE2 for both SARS2 WT and OV as an example [33]. Y505H, N501Y, Q493R OV mutations clearly form more pairs and which are relatively stronger with ACE2 than their WT counterparts, indicating that they have potential to increase ACE2 binding. In contrast, the E484A mutation loses the interaction with K31 of ACE2, reducing ACE2 binding. Furthermore, even though G446S and G496S yield the same number of pairs in both strains, their AABP strengths differ. With a few exceptions, these observations appear to be in strong agreement with experimental and computational data [34,108,109,110]. The RBD Omicron mutations may have an impact on the interactions of the conserved RBD residues with ACE2, particularly those adjacent to the mutations whose biological environments have changed because of the mutations. Indeed, Y449 and T500 adversely influence the interactions with ACE2, while the opposite occurs for L455, A475, and Y489 unmutated residues [33]. The same trend emerges in Figure 5b for RBD–ACE2 complexes of WT and OV, but with more nuances since the whole Omicron RBD BA.1 has 15 mutations instead of 10. Obviously, there are some distinct differences between the AABP in RBM–ACE2 and RBD–ACE2 in terms of pairing and strength, especially for K417N, N440K, G446S, S477N, and N501Y. Because K417N is located outside the RBM, its pair is not included in the RBM–ACE2 model. Our calculations point to the conclusion that N417K results in losing the strong or weak pairs with D30 and H34 of ACE2. This observation is consistent with structural and computational studies [32,111]. For the differences in N440K, G446S, S477N, and N501Y, we speculate the following: First, the RBD–ACE2 is a more realistic model than RBM–ACE2, so the intramolecular RBD interactions are different due to many factors, including the impact of extra mutations, the degree of freedom, etc. Second, the OV initial structures for both models are not the same, implying different configurations of these residues between the RBD and the RBM.

Now, we turn our discussion to some interesting observations about the drift of Omicron mutations towards a more positive charge. Except for Y505H, all mutations on the RBM–ACE2 model tend to have a positive PC*. Again, the PC* is determined by adding the partial charges of all the AAs in the AABPU, including the PC of the mutated site and the other AAs with which it interacts. Q498R exhibits the highest increase in the PC* [33]. This increased behavior in PC* is correlated with a shift toward positive charge in partial charge per amino acid (PC^AA^) from the only mutated site, with eight out of ten mutated sites exhibiting this trend. Similarly, 10 out of 15 Omicron mutations in the RBD–ACE2 model have changed PC* toward being positively charged [31]. These are S371L, S373P, S375F, G446S, S477N, T478K, E484A, Q493R, N501Y, and Y505H. Interestingly, the shift toward positive PC* is significant in the AABPU of G446S, S477N, T478K, Q493R, N501Y, and Y505H, indicating major changes in their charge density distributions and electrostatic potentials. For most mutations, the PC^AA^ per mutated site acts similarly to PC*. However, the characteristics of PC* and PC^AA^ for the Q498R mutation is markedly different.

Importantly, the Omicron mutations resulted in a more positive partial charge distribution of RBD which in turn shifts the charge of the OV S-protein toward more positive than the WT S-protein. This shift in charge distributions has many implications. First, it can interact more attractively with the negatively charged ACE2, enhancing the binding affinity of the OV RBD–ACE2 complex compared with the WT strain. This factor is assigned as the main source of increase in infectivity of OV [34,106,110]. Second, it can electrostatically repel the positive charge of antibodies, conferring immune resistance [112]. Finally, it makes the S-protein more sensitive to low-pH-induced conformational changes, promotes an adaptation that uses the low-pH endosomal entry pathway, and/or increases virus entry in the lower pH environment of the upper airway [90].

### 3.3. Challenges and Opportunities in First Principles DFT Calculation for SARS-CoV-2

This paper provides several computational advances, allowing us to address several fundamental questions. First, we carried out an unprecedented large-scale DFT calculations on the S-protein of SARS-CoV-2; second, we outlined a roadmap for future computational research in the context of biomolecular systems generally, and prevention of viral transmission specifically. In the future, the current computational techniques based on fundamental quantum chemical calculations at an atomistic level can be expanded further to study additional details of viral mutation and proliferation and shed additional light onto the mechanism of infection. The approach that we advocate goes beyond the purely AA-sequence-based bioinformatics and introduces functional and interactional units, the AABPUs, that can expand the parameter space for performance optimization.

The most well-known drawback of density functional theory (DFT) calculations is the one stemming from the size of the system. However, there has been solid progress in recent years balancing time and cost considerations. The Ab initio fragment molecular orbital (FMO) approach divides big molecules into small fragments and calculates molecular orbitals in each fragment to determine the properties of the entire system [113]. Recently, the FMO approach was implemented to study the SARS-CoV-2 S-protein interactions with the ACE2 and antibodies [114,115,116] based on the *divide and conquer strategy*, dividing the S-protein into several domains/regions of interest and then analyzing each of them separately. With this approach, we were able to conduct single DFT calculations on domains composed of up to 5000 atoms [25,26,27,29,30,31,32,33,34,35].

## 4. Discussion

Protein–protein interaction in biomolecules is crucial and engender further cascades of events leading to significant outcomes, such as viral entry into the host cell. Using an ab initio quantum chemical approach, these interactions can be studied on an atomistic level. In our approach, we conceptualized and calculated the AA bonding by introducing the AABP, identifying the interacting units and quantifying their interaction strength, used as a proxy for bonding strength determination. The use of AABP can be further implemented for other systems in order to study protein–protein interactions in 3D protein structures at the ab initio quantum chemical level. In these cases, the AABPU can lead to better understanding of protein structural interactions based on parameters such as its partial charge, its shape and size, and is able to identify important mutational effects on the S-protein.

Partial charge (PC) can be considered as an important parameter to account for the mutational drift associated with the new VOCs. The change in PC of AABPU can identify new environments created by mutation and quantify the mutational drift, with the capability to provide a rationale for emerging behaviors of the newer variants, such as high infectivity and transmissibility of OV. Our calculated partial charge has identified and quantified the continuous growth in the number of positively charged AAs in the solvent-exposed regions of the S-protein and its ACE2-binding sites, as well as in the RBD epitopes that are targeted by drugs and therapeutic antibodies.

The quantified PC obtained from DFT can be used furthermore to calculate the electrostatic interaction using, e.g., the Delphi software [32,78]. In addition, the PC values can be used in MD simulations to improve their accuracy. MD standardly uses a fixed PC based on a priori force fields, and these PCs cannot describe the changes in the PCs associated with breaking and forming of covalent bonding between atoms. The PC values based on ab initio quantum chemical calculations can be fed into the force-field parameters for an accurate electrostatic interaction prediction.

In conclusion, we carried out and analyzed an unprecedented large-scale DFT calculation on the S-protein of SARS-CoV-2 and outlined a roadmap for future computational research for biomolecular systems in general. The techniques introduced in this approach can be expanded further to study the details of virus infection and proliferation processes by providing a solid understanding of the underlying molecular mechanisms based on fundamental quantum chemical calculations at an atomistic level.

## Figures and Tables

**Table 1:** Comparison of AABP units of NTD sites between wild type (WT), Delta variant (DV), and Omicron variant (OV).

Models	Total AABP	NN AABP	Non-local AABP	AABP from HB	No. of NL AAs
WT T19	1.121	1.053	0.068	0.124	1
DV R19	0.959	0.958	0.001	0.024	3
WT G142	1.147	1.014	0.133	0.154	10
DV D142	1.030	0.940	0.090	0.100	4
WT E156	1.208	0.959	0.249	0.229	10
DV G156	0.918	0.465	0.453	0.029	4
WT A67	1.064	0.989	0.075	0.092	7
OV V67	1.046	0.965	0.081	0.093	10
WT T95	1.053	0.998	0.055	0.075	9
OV I95	1.079	1.008	0.072	0.084	11
WT G142	1.147	1.014	0.133	0.154	10
OV D142	1.265	1.007	0.257	0.251	9
WT N211	1.028	1.028	0.031	0.089	3
OV I211	0.949	0.944	0.005	0.031	5

Background color: blue (WT), green (DV), and yellow (OV).

**Table 2:** Comparison of AABP units between wild type (WT), top 2 mutations for Delta variant (DV) and 16 mutations for Omicron variant (OV) BA.1 in the RBD-SD1 domain from reference [31]. AABP is in unit of electrons (e^−^).

Models	Total AABP	NN AABP	NL AABP	No. of HBs (HB AABP)	No. of NL AAs	Volume (Å^3^)	Area (Å^2^)	PC* (e^−^)
WT L452	1.022	0.978	0.045	31 (0.061)	9	1641.0	1048.0	−0.074
DV R452	1.019	0.976	0.043	26 (0.064)	8	1549.0	972.8	0.849
WT T478	1.044	1.043	0.001	9 (0.022)	1	335.1	333.5	0.005
DV K478	1.219	1.217	0.002	13 (0.136)	3	571.1	497.8	1.022
WT G339	1.016	0.993	0.023	11 (0.052)	3	652.2	570.6	−0.340
OV D339	1.196	1.154	0.042	14 (0.063)	4	807.7	634.1	−1.357
WT S371	0.918	0.888	0.030	20 (0.051)	5	854.7	680.5	−0.147
OV L371	0.945	0.928	0.017	21 (0.040)	3	608.7	532.5	−0.162
WT S373	0.941	0.920	0.021	14 (0.052)	3	633.6	543.4	−0.075
OV P373	0.999	0.992	0.008	16 (0.031)	3	764.9	623.2	−0.084
WT S375	0.944	0.916	0.028	11 (0.058)	4	808.4	642.2	−0.026
OV F375	0.926	0.917	0.009	13 (0.037)	7	1331.0	941.1	0.076
WT K417	1.216	1.013	0.203	19 (0.203)	7	1195.0	827.5	0.153
OV N417	1.066	1.017	0.048	14 (0.069)	6	987.4	697.0	−1.473
WT N440	0.985	0.981	0.005	12 (0.037)	3	584.2	467.3	−0.802
OV K440	0.983	0.978	0.005	14 (0.037)	4	825.4	645.6	0.148
WT G446	0.912	0.910	0.002	10 (0.038)	2	473.8	403.3	0.907
OV S446	1.038	0.979	0.059	14 (0.091)	2	530.4	443.5	1.843
WT S477	0.964	0.958	0.006	12 (0.039)	2	440.7	383.3	0.100
OV N477	1.157	1.156	0.001	11 (0.151)	2	507.3	428.0	1.097
WT T478	1.044	1.043	0.001	9 (0.022)	1	335.1	333.5	0.005
OV K478	1.214	1.212	0.002	13 (0.139)	3	594.9	509.1	1.045
WT E484	1.040	0.927	0.114	19 (0.124)	4	828.5	633.4	−0.967
OV A484	0.934	0.932	0.002	13 (0.030)	3	513.8	452.9	−0.081
WT Q493	1.060	0.973	0.087	19 (0.106)	6	1220.0	786.3	0.497
OV R493	1.165	1.165	0.194	32 (0.200)	9	1739.0	1034.0	−0.498
WT G496	0.975	0.944	0.031	11 (0.062)	4	834.4	691.4	−0.285
OV S496	0.994	0.938	0.055	12 (0.076)	4	964.4	755.0	0.657
WT Q498	1.120	1.073	0.047	25 (0.054)	10	1376.0	894.1	1.013
OV R498	1.179	1.056	0.123	30 (0.126)	11	1648.0	1078.0	2.059
WT N501	1.120	1.073	0.047	27 (0.054)	10	1063.0	802.3	0.022
OV Y501	1.034	0.942	0.092	21 (0.104)	6	1089.0	797.4	0.752
WT Y505	1.058	0.974	0.084	17 (0.104)	6	983.1	714.7	0.156
OV H505	0.998	0.953	0.045	15 (0.069)	7	1188.0	859.7	0.283
WT T547	1.033	0.977	0.056	20 (0.079)	4	738.6	527.8	0.150
OV K547	0.994	0.977	0.016	21 (0.042)	4	773.5	584.4	1.100

Background color: blue (WT), green (DV), and yellow (OV).

## Data Availability

The data can be provided on request to the corresponding author.
